# Thermal variability and diet interaction as driver of developmental overwintering in *Drosophila buzzatii*

**DOI:** 10.1038/s41598-025-25689-8

**Published:** 2025-11-07

**Authors:** Lucas Kreiman, Valeria Careaga, Eduardo M. Soto, Daniela Peluso, Esteban Hasson, Pablo E. Schilman, Julián Mensch

**Affiliations:** 1 Laboratorio de Evolución, Departamento de Ecología, Genética y Evolución, Facultad de Ciencias Exactas y Naturales, Universidad de Buenos Aires, Buenos Aires, Argentina; 2 Instituto de Ecología, Genética y Evolución de Buenos Aires (IEGEBA), CONICET-Universidad de Buenos Aires, Buenos Aires, Argentina; 3 Departamento de Química Orgánica, Facultad de Ciencias Exactas y Naturales, Universidad de Buenos Aires, Buenos Aires, Argentina; 4 Unidad de Microanálisis y Métodos Físicos en Química Orgánica (UMYMFOR), CONICET-Universidad de Buenos Aires, Buenos Aires, Argentina; 5 Laboratorio de Biología Integral de Sistemas Evolutivos (BISE), Departamento de Ecología, Genética y Evolución, Facultad de Ciencias Exactas y Naturales, Universidad de Buenos Aires, Buenos Aires, Argentina; 6 Laboratorio de Ecofisiología de Insectos, Departamento de Biodiversidad y Biología Experimental, Facultad de Ciencias Exactas y Naturales, Universidad de Buenos Aires, Buenos Aires, Argentina; 7 Instituto de Biodiversidad y Biología Experimental de Buenos Aires (IBBEA), CONICET-Universidad de Buenos Aires, Buenos Aires, Argentina

**Keywords:** Viability, Developmental acclimation, Homeoviscous adaptation, Fatty acids, Chill coma recovery time, Ecophysiology, Evolutionary ecology

## Abstract

Global warming influences organisms through direct and indirect effects on abiotic and biotic factors, such as temperature and food availability. This study examines how developmental diet and thermal regime influence fitness and thermal tolerance in *Drosophila buzzatii*, a cactophilic fly with a broad thermal tolerance, and a model organism in evolutionary ecology. Using a factorial design, we reared flies at 10 °C and 25 °C, with and without daily thermal fluctuations, on two natural cactus-based diets and a standard lab diet. We measured viability, development time, wing length, ovariole number, chill coma recovery time and fatty acid composition. We found synergistic effects between cactus diet and thermal daily fluctuations, overcoming the complete loss of viability observed at 10 °C and mitigating the negative effects shown at 25 °C. In addition, flies reared at a low fluctuating temperature had greater cold tolerance and a higher unsaturated-to-saturated fatty acid ratio, supporting the homeoviscous adaptation hypothesis. These findings suggest *D. buzzatii* may overwinter as larvae and pupae in temperate areas and highlight the importance of diet-thermal interactions in shaping insect responses to climate change.

**Supplementary Information:**

The online version contains supplementary material available at 10.1038/s41598-025-25689-8.

## Introduction

Global warming due to climate change is expected to have devastating effects on living organisms, biodiversity and ecosystem stability^1^. Ectotherms, whose body temperature depends on external sources of heat, are thought to be especially vulnerable to global warming^2,3^. Additionally, rising temperatures caused by climate change have consequences over many other environmental factors^4^. For instance, rising temperatures have been reported to negatively affect plant productivity^5^ and nutritional content, disturbing the trophic network as a whole^6^. This is important for insect biology, as the class is ecologically linked to plants in many ways^7^. On one hand, over 290,000 insect species serve as pollinators for more than 250,000 entomophilic angiosperms species, which account for almost 70% of plant diversity^8^. On the other hand, insect herbivory imposes a limit to plant population growth^9^. In response, plants have evolved several strategies against herbivory, such as the production of toxic allelochemicals, triggering the evolution of species-specific detoxification pathways in insects^10,11^. Some holometabolous species have even incorporated the otherwise toxic chemistry of its host plants to its lifestyle.

Cold tolerance is one of the main drivers that modulate the limits of species ranges and distributions^12,13^. In effect, terrestrial insects in temperate and polar environments may spend more than half their lives overwintering^14^. In such seasonal environments overwintering performance has been shown to be one of the most important factors controlling insect abundance and therefore inter-annual population persistence. Overwintering strategies are diverse. While some insect species overwinter as adults others do it in pre-adult stages, though there are some cases of mixed strategies (e.g. overwintering as larvae, pupae and adults in any combination)^15^. Likely, there is no general rule regarding which ontogenetic stages present stronger cold tolerance^16^. While some species show more tolerance to cold at early stages of development, others are stronger at late stages or at adult stages. Notably, exposure to temperatures lower than population- or species-specific thresholds can result in mortality or sub-lethal cold injuries^17^. Understanding the biotic and abiotic drivers that influence overwintering performances can shed light into insect capacity to tolerate long-term cold exposure.

Low temperature during development can induce phenotypic changes that improve adult cold tolerance. For example, developmental acclimation is associated with substantial changes in lipidic composition favoring unsaturated fatty acids in relation to saturated ones^18^, commonly known as the homeoviscous adaptation hypothesis^19^. The rationale is that unsaturated fatty acids, due to its complex geometry, can maintain membrane fluidity at low temperatures^18^ and thus protect the functionality of membrane elements such as enzymes and ion channels^20^. Other effects of low temperature developmental acclimation are related to overwintering performance as a result of changes in adult body size and pigmentation^21^. For example, low temperature during development can also induce larger and darker adults, which in turn improve adult cold tolerance leading to a high overwintering performance^22^.

Developmental diet also has a determining effect of the thermal tolerance of adult flies, measured as survival to cold exposure, fecundity, critical thermal minimum^23^ and chill-coma recovery time^24,25^. Lipids in particular play a key role; a lipid-enriched diet shortens chill-coma recovery time^25^, and a cholesterol-enriched diet improves *Drosophila melanogaster* survival after chill injury^26,27^. As diet affects insect thermal tolerance^28,29^, some insect species have been reported to switch diet in response to low temperatures. At 15 °C, *D. melanogaster* switches preference for oviposition site from protein-rich diet to plant-based diet, improving cold tolerance and longevity of its offspring^30^.

Diet and temperature interact in complex ways and affect insects’ fitness, especially during development^31^. For example, a developmental diet can modulate responses to temperature, such as the sign of temperature-body size correlation^32^ and the maximum of thermal performance curves^33^. In the cactophilic fly, *Drosophila buzzatii*, nutritional stress can affect the predictability of the relationship between temperature and morphological traits such as wing and thorax length^34^. Diet-by-temperature interactions during development also have strong effects on several other fitness-related traits. In *D. melanogaster*, temperature during development along with Protein: Carbohydrate proportion in breeding diet can alter developmental rate, body size^35^, viability^36^, and wing area^35^. Developmental diet requirements have been shown to be affected by daily thermal fluctuations. For example, a daily fluctuation of ± 5 °C at 25 °C lowers Protein: Carbohydrate proportion needed to maximize viability in *D. melanogaster*. In contrast, the same range of fluctuation at 28 °C has the opposite effect^37^. Daily thermal fluctuations are often overlooked as a factor in experimental designs, as thermal experiments are usually performed at constant temperatures^38^. However, with rising temperatures, daily fluctuations are also expected to increase^39^. These changes in temperature regimes have been shown to critically affect several fitness-related traits in insects^40–42^. While the impact of thermal variability is commonly studied at high temperatures (i.e. summer-like conditions), little is known about how thermal variability can affect insect overwintering strategies under climate change^43^.

The *repleta* species group is a monophyletic lineage of New World *Drosophila* that includes species that acquired a cactophilic lifestyle, i.e. breed and feed on decaying cactus tissues. Some species have been widely studied due to the tight ecological and evolutionary association with cacti and the microbial community involved in decomposition, known as the cactus-microorganism-*Drosophila* system^44,45^. For example, *D. buzzatii*, a member of the *buzzatii* species cluster, is one of the South American branches of the *repleta* group^46^. This species exploits primarily cacti of the genus *Opuntia* and, alternatively, columnar species of the genera *Cereus* and *Trichocereus*^44^. Its occurrence closely matches cactus species abundance, both in its native^47^ and exotic distributions^48,49^. Besides being the subject of many ecological and evolutionary studies, due to its close association with cacti, *D. buzzatii* has also been of interest to climate change biologists due to its broad thermal tolerance^50,51^. Its distribution spans over regions of highly diverse weather and seasonality (Figure S1) and high and low altitude lands^52^. Accordingly, when compared to other species of the *repleta* group, *D. buzzatii* can be described as tolerant to both cold^53–55^ and heat^50,56,57^. Most studies in this species have been performed under constant temperatures^34,53,58–60^ or alternating between two constant temperatures to account for daily thermal variation^61–63^. In addition, the interaction between developmental diet and thermal fluctuation has been only studied so far in the cosmopolitan and polyphagous model organism *D. melanogaster*^35,37^, however, it is yet to be tested in a specialist, ecologically-relevant model species, such as *D. buzzatii*. In this context, we previously hypothesized that annual persistence of *D. buzzatii* in regions where winter average temperatures fall below lower thermal thresholds could be due to pre-adult stages developing in rotten cacti taking advantage of daily thermal fluctuations^53^.

The aim of this paper is to study the combined effect of developmental diet, temperature, and daily thermal fluctuations on fitness- and cold tolerance-related traits in *D. buzzatii*. To this end, we run a factorial design of temperature and diet, using both semi-natural (cactus-based) and laboratory diets across two constant and two fluctuating thermal regimes. For the fluctuating regimens, we implemented a model that closely follows daily temporal variation^64^ using parameters derived from winter temperatures of the collection site. This experimental design would allow us to understand whether *D. buzzatii* is capable of overwintering in pre-adult stages.

## Methods

### Flies stock

We studied five isofemale lines (‘lines’ hereafter) of *D. buzzatii* derived from collections in San Agustín del Valle Fértil, Argentina (31°7′4.08″ S, 67°40′42.96″ W, see Fig. S1) in March 2014 ^60^, as a way to take genetic variability in consideration. Flies were caught by net sweeping on fermented banana bait traps disposed near decaying cacti. Upon arrival at the field lab, flies were separated by sex and placed in individual vials. Species identification was accomplished by the inspection of male genitalia (aedeagus). Prior to the experiments, lines were maintained under controlled humidity (*~* 70%), photoperiod (12:12), and temperature (*~* 25°C) and fed with instant mashed potato medium hydrated with a water solution of the antifungal Nipagin (p-hydroxybenzoic acid methyl ester) and yeast as protein source (laboratory diet)^54^.

### Rearing of experimental flies

A description of the experimental design can be found in Fig. 1. We tested the performance of flies raised in media prepared with its main natural host, *Opuntia sulphurea*, and the alternative host *Trichocereus terscheckii*. For the preparation of cactus media, pieces of cactus were ground in a blender, weighted and misxed baker’s yeast in a 99:1 proportion. About 8 g of media were poured into each vial, which were then sterilized in an autoclave. Nutritional composition of diets can be found in Table S1.

**Figure:**
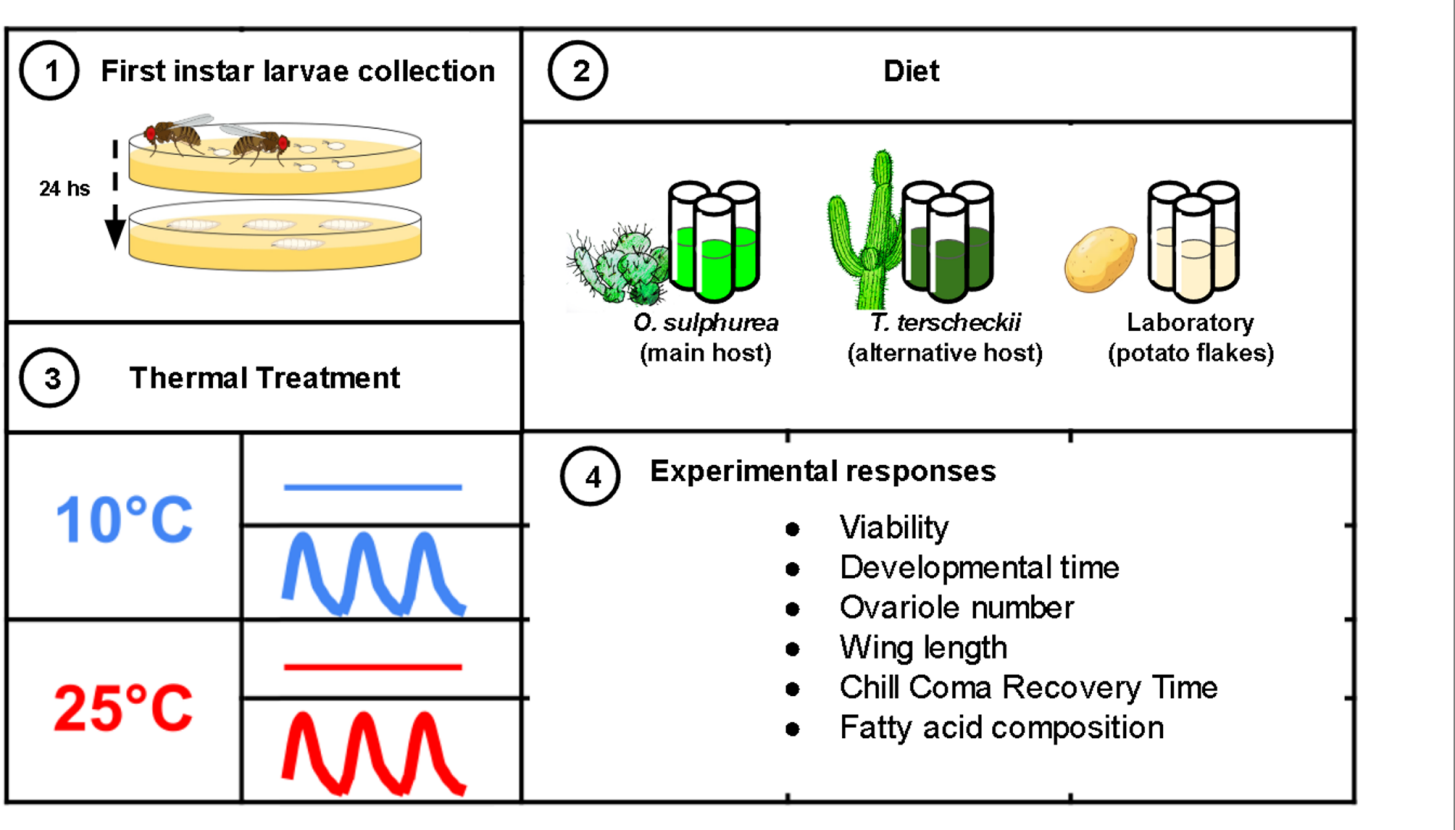
**Fig. 1**Flowchart of the experimental design. (1) First, adult *D. buzzatii* were allowed to mate and lay eggs on petri dishes containing agar, and yeast to stimulate ovipositing^65^. (2) Batches of 40 first-instar larvae were transferred to culture vials containing either a semi-natural diet prepared with cactus or the laboratory diet (instant mashed potato medium hydrated). (3) Samples were randomly assigned to an incubator set to a specific temperature program. Total numbers of replicates by line are mentioned in Table S4. (4) Viability and developmental time were recorded, and ovariole number, wing length, chill coma recovery time, and fatty acid composition were measured.

**Fig. 2:**
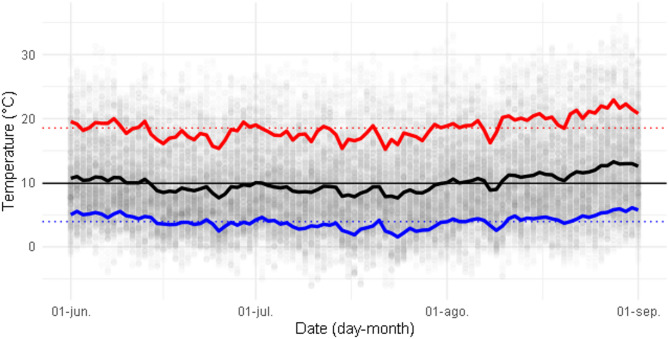
Winter temperatures at the collection site (Valle Fértil, Argentina). Hourly temperature data recorded during winters (June 1 to September 1) from 2001 to 2024 at the site’s coordinates with a one-half arc degree longitude by one-half arc degree latitude resolution. Each point represents a temperature measurement obtained from the NASA POWER API database, using the nasapower R package^67^, with darker hues indicating data overlap. The red and blue lines show the mean daily maximum and minimum temperatures, respectively, while the black line represents the mean daily temperature. Dotted lines indicate mean maximum and mean minimum value, and the straight black line represents the general mean, 9.91 °C.

Four temperature treatments were used, two constant regimes at 10 °C and 25 °C; and two fluctuating ones at 10 ± 6 °C and 25 ± 6 °C, according to the Parton-Logan model of daily fluctuation^42,64^. The low-temperature mean of 10 °C was chosen as it mimics the average temperature during the coldest months (June to August) of a 24-year record (2001–2024) at the collection site (Fig. 2). The mean daily minimum and maximum temperatures were 3.9 °C and 18.5 °C, respectively (Fig. 2). However, as cactus tissue is known to buffer environmental temperatures^66^, a temperature range from 4 °C to 16 °C, with a mean of 10 °C, was chosen. The high-temperature mean was set at 25 °C, maintaining the same thermal range as the low-temperature treatment (± 6 °C).

### Life history traits

A total of 711 vials were run, with 8 to 16 vials per combination of line, diet, and temperature. Vials were revised daily to check for adult emergence. Once emerged, adults were removed from the vials. Viability was calculated as the percentage of adults emerged in each vial relative to the 40 initial larvae. Developmental time was measured as the number of days elapsed from first instar larvae to adult emergence. The assay was finished when all adults emerged or after several weeks without emergence.

### Wing length and ovarioles number

Body size is generally considered as an important indicator of fitness in insects^68^, and wing length as a proxy of body size, particularly in cactophilic *Drosophila*^69,70^. Ovariole number correlates positively and significantly with total reproductive output^71^, particularly in Drosophilidae^72^, and as such is a reproductive component of fitness. Immediately upon emergence, flies have immature, small ovaries and folded wings. Thus, newly emerged adults were separated by sex and transferred to vials with fresh Laboratory diet at *~* 25 °C for seven days, allowing females to reach ovarian maturation^60^. Wings were removed and mounted using DPX mounting adhesive. Digital images of the two wings of each individual were obtained using a Leica M205 A stereo microscope equipped with a Leica DMC 2900 digital camera. Wing length was measured as the distance in millimeters between the beginning and the end of vein 3 (see Fig. S2) using Leica Application Suite (LAS) software V4.13. Ovarioles dissection was performed following the protocol described in Peluso et al., 2016 ^60^. Briefly, ovaries were pulled away from the abdomen in the posterior direction using forceps, and dyed with methylene blue. Then ovarioles were stained and separated for counting (Fig. S3).

### Chill coma recovery time (CCRT)

Chill coma recovery time has been extensively studied as a measure of cold tolerance in insects^73^. After 6–8 days at 25 °C, flies were transferred without anesthesia to empty vials immersed in an ice-water (i.e. 0 °C) in a polystyrene box placed inside a refrigerator at 4 °C. This setup prevented ice from fully thawing and kept icy-water at 0 °C for 12 h. Flies were allowed to recover at 25 °C. Recovery from chill coma was individually measured as the time (in seconds) elapsed until flies could stand up on their legs^74^.

### Fatty acid profiling

Upon emergence individuals were snap-frozen in liquid nitrogen. Samples were stored at − 80 °C until further processed. Based on pilot studies, 20 individuals per replicate and 3–4 replicates per experimental group were assigned. For these analyses, only two diets (*O. sulphurea* and Laboratory) were used as no significant differences were found between cactus diets in the CCRT assays. The pool of 20 individuals was weighed together before analysis. A homogenate of 20–45 mg per sample was prepared from these individuals. Approximately 3.5 to 10 mg of lipids were recovered. Fatty acid methyl esters (FAMEs) of total lipids were prepared by reaction with 4% HCl in CH_3_OH at 70 °C for 2 h. After cooling, a drop of water was added and the FAMEs were extracted with 0.5 mL of dichloromethane three times. The organic phase containing FAMEs were analyzed by gas chromatography (CG-FID) on a Focus GC (Thermo Finnigan Corporation), equipped with an Innowax capillary column (Agilent, 100% polyethylene glycol, 30 m length, 0.25 mm i.d., 0.5 μm film thickness), as described in Gomez Ribot et al. ^75^ and supplementary material. Quantification was performed by comparing the peak area percentages on the chromatogram with that of the internal standard of known weight (methyl nonadecanoate, Sigma-Aldrich Co.). Along with these individuals, fatty acid compositions of the experimental diets were also analyzed.

### Statistical analyses

All analyses were performed employing R version 4.3.0 ^76^ and RStudio (“Cherry Blossom”, v2023.3). Data visualization was done via ggplot2 ^77^. Univariate analyses were implemented using generalized linear mixed models (GLMMs) constructed with the glmmTMB package^78^ and validated with DHARMa^79^. Our goal was to investigate the effects of diet, temperature, and daily thermal fluctuation on fitness, and their interactions. However, not all interactions could be tested because rearing the flies at 10 °C on any diet and at 10 ± 6 °C on the laboratory diet resulted in null viability, and consequently in perfect separation (i.e. rank deficiency). To address this problem, we introduced a new variable by merging diet and temperature levels, defining eight experimental groups: 10 ± 6 °C with *O. sulphurea* or *T. terscheckii*; 25 °C and 25 ± 6 °C with *O. sulphurea*, *T. terscheckii*, or Laboratory diet. A separated factorial analysis was performed exclusively over the 25 °C and 25 ± 6 °C data, as this subset constituted a fully ranked matrix. The number of replicates can be found in Table S4. GLMMs were used to evaluate the effect of the experimental group on the response variable, incorporating ‘line’ as a random effect. We assessed the significance of the line factor by comparing each model with a reduced one without the random effect. In cases of heteroskedasticity, variance was modeled accordingly. Individual fatty acid concentrations were assumed to follow a normal distribution, while ovariole number was modeled using a Poisson distribution^80^. Developmental time and CCRT were fitted to a lognormal distribution, whereas wing length was modeled with a Gamma distribution using a logarithmic link function. Lastly, viability was analyzed using a zero-inflated binomial model, including an observation-level random effect (OLRE) to account for overdispersion^78,80^. Wald’s χ^2^ test was performed via the Anova() function of the car package^81^, and then the emmeans package^82^ was used for *post hoc* multiple mean comparisons across groups. The factoextra package^83^ was used to conduct a Principal Component Analysis (PCA) on a data matrix containing all 11 fatty acid values, applying a singular value decomposition (SVD) approach.

## Results

### Life history traits

**Fig. 3:**
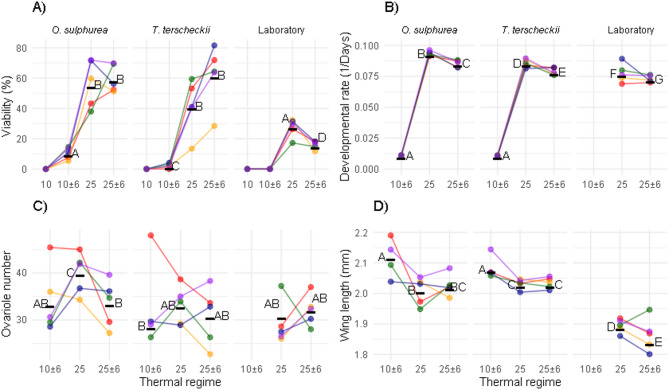
Reaction norms of viability, developmental rate, ovarioles number and wing length of the experimental groups. Colors indicate different isofemale lines, columns indicate diet levels, and the horizontal axis represents developmental temperature (mean constant or mean ± daily thermal variation) in °C. Points represent the mean value of each isofemale line and the horizontal black line represents the mean value of the experimental group, which can be found in Table S5. Different letters denote significant differences (*p* < 0.001) derived from *post hoc* comparisons. (**A**) Viability, measured as the percentage of first-instar larvae that reached the adult stage. (**B**) Developmental rate measured as 1/days elapsed from first-instar larvae to adult emergence. (**C**) Ovariole number per female (**D**) Wing length (mm), distance between start and end of vein 3.

At constant 10 °C no individuals could complete development in any diet, and a modest number of individuals reared at 10 ± 6 °C reached adulthood only in the semi-natural diets, i.e., 10.4% in *O. sulphurea* and 2.1% in *T. terscheckii* (Fig. 3A). Experimental groups showed significantly differences in viability (χ^2^ = 382.43, *p* < 0.0001, Fig. 3A). A separated analysis of the 25 °C and 25 ± 6 °C dataset revealed a significant Diet-by-Daily thermal fluctuation interaction (χ^2^ = 57.52, *p* < 0.0001, Table S6), consistent with the *post hoc* comparisons shown in Fig. 3A. Daily thermal fluctuations around 25 °C preserved high viability only in flies reared on semi-natural diets, while these fluctuations significantly reduced the viability of juveniles raised on the Laboratory diet from 28.3 ± 17% to 15.7 ± 11.5% (Fig. 3A). Flies reared at 10 ± 6 °C had long developmental times (90.9 ± 6.1 days, Fig. 3B). Daily fluctuations at 25 ± 6 °C increased developmental time in all diets (Fig. 3B). The crossing of the reaction norms for viability and developmental time further indicated interplay between diet and thermal regime (Fig. 3). The line factor was significant for both viability (χ^2^ = 7.29, *p* = 0.0007) and developmental time (χ^2^ = 37.25, *p* < 0.0001).

### Wing length and ovarioles number

Flies reared at 10 ± 6 °C had larger wings (2.11 ± 0.07 mm, Fig. 3D), but did not differ in ovariole number from most treatments at 25 °C and 25 ± 6 °C (32.0 ± 7.9 ovarioles, Fig. 3C). As with viability, daily fluctuations at 25 ± 6 °C significantly reduced wing length in flies reared on laboratory diet, but had no effect when combined with semi-natural diets (Fig. 3D). In addition, some flies reared at 10 ± 6 °C showed wing malformations, including both incomplete and ectopic veins (Fig. S4). Interplay between diet and temperature could also be observed by the crossings of the reaction norms for ovarioles number and wing length (Fig. 3). We found significance for the line factor in for both ovariole number (χ^2^ = 33.01, *p* < 0.0001) and wing length models (χ^2^ = 64.73, *p* < 0.0001).

### Chill coma recovery time

**Fig. 4:**
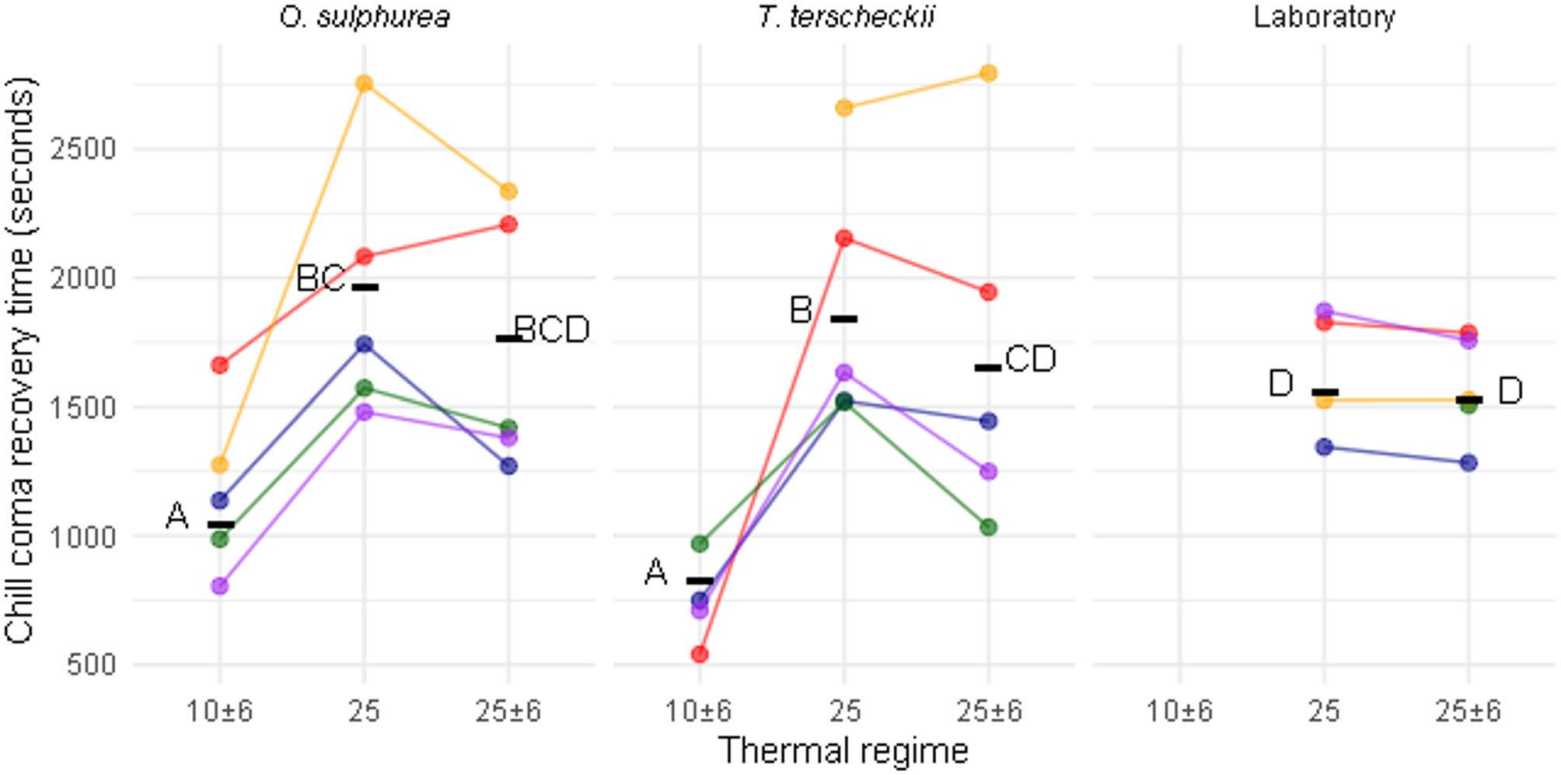
Chill Coma Recovery Time of the experimental groups. Recovery time was recorded at 25 °C after a 12 h exposure at 0 °C. Columns indicate diet levels, colors indicate different isofemale lines and the horizontal axis represents the developmental temperature (mean constant or mean ± daily thermal variation) in °C. Points represent the mean value of each isofemale line and the horizontal black line the mean value of each group, which can be found in Table S5. Different letters denote significant differences (*p* < 0.001) derived from *post hoc* comparisons.

Figure 4 shows the differences in chill coma recovery time across experimental groups. Flies reared at 10 ± 6 °C recovered significantly faster (χ^2^ = 111.18, *p* < 0.0001, Fig. 4) from chill coma (~ 16 min) than flies reared at 25 °C, with or without daily fluctuations (~ 27 min). Flies reared at 25 °C in the Laboratory diet had significantly faster recovery than those reared in the semi-natural diets (Fig. 4). However, flies reared at 25 ± 6 °C showed no significant differences across diets (Fig. 4). Line resulted significant (χ^2^ = 95.0, *p* < 0.0001) and an interplay between diet and thermal regime was indicated by the crossing of reaction norms (Fig. 4).

### Fatty acid profiles

Eleven fatty acids were identified in adults’ fatty acid fraction, of which the most abundant were C16:0 (Palmitic), C16:1 (Palmitoleic), C18:1 (Oleic) and C14:0 (Myristic) (see Table S2 for details). While abundant in adult flies, C14:0, C12:0, C12:1 and C14:1 were absent from both diets (Table S3).

**Fig. 5:**
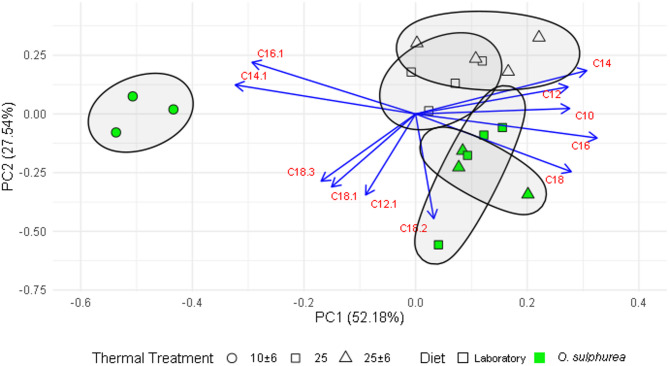
Principal Component Analysis (PCA) of fatty acid profiles across experimental groups. Colors indicate diets, and shapes represent thermal treatments. Blue arrows illustrate the contribution of each variable (fatty acid) to the first two principal components, which together explains 79.7% of total variance. Ellipses enclose replicates by experimental groups (Diet-Thermal treatment combination) and were calculated using Khachiyan’s algorithm^84^.

The first two principal components of the PCA accounted for 79.72% of total variance (Fig. 5). PC1, which is composed mainly by the monounsaturated fatty acids (specially, C16:1 and C14:1) significantly separated (χ^2^ = 275.49, *p* < 0.0001) samples reared at 10 ± 6 °C from those reared at 25 °C and 25 ± 6 °C. In addition, PC2 discriminates high temperature groups by diet: flies reared in the semi-natural diet had higher values of C12:1 and unsaturated 18-carbon fatty acids than those reared in the Laboratory diet (χ^2^ = 57.08, *p* < 0.0001). PC2 could not discriminate between the low temperature (10 ± 6 °C) group from high temperature (25 °C and 25 ± 6 °C) groups (Fig. 5).

Univariate analyses further discriminated the low fluctuating temperature group (10 ± 6 °C) from flies reared at both 25 °C and 25 ± 6 °C. The low temperature group was significantly enriched in unsaturated, particularly mono-unsaturated fatty acids (namely, C14:1 and C16:1), and it had twice the UFA/SFA ratio (Table S2).

## Discussion

Due to its influence on both biotic and abiotic factors, as well as their interactions, climate change has multifaceted effects on species. In this study, we showed how diet and thermal regimes interact in a non-additive way affecting several traits. We found a strong interplay between thermal regime and diet during development, influencing life history, morphological and reproductive traits, as well as thermal tolerance and fatty acid composition in the cactophilic fly *D. buzzatii*. This species appears to be adapted to both fluctuating temperature and cactophilic lifestyle, which is consistent with the fact that it evolved in what is known as the ‘South America’s dry diagonal’, a chain of environments exposed to high thermal amplitude and seasonality^85^, rich in cactus diversity^47^.

### Life history traits

Daily thermal fluctuations at 25 °C had a severe negative effect on developmental time in all diets and reduced viability of flies reared in the laboratory diet, but had no effect on flies reared in cactus diets. However, the combined effect of daily thermal fluctuations and cactus diet allowed *D. buzzatii* to overcome the complete loss of viability observed at constant temperature below its lower thermal threshold, which seems to be around 10 °C. This is a relevant finding, as it suggests that, in temperate regions, *D. buzzatii* may have the ability to overwinter not only as virgin or inseminated females under cold-induced reproductive arrest^53^, but also as larvae or pupae with slow development induced by low temperatures. However, it is important to point out that development at low temperatures involves severe fitness costs, such as low pre-adult survival and long developmental time, averaging over 90 days.

### Wing length and ovariole number

Flies reared at 10 ± 6 °C have longer wings, but also showed cases of wing morphology abnormalities. A similar syndrome has been documented as a sign of developmental stress in response to a diet of the cactus *T. terscheckii*, which is rich in alkaloids^59^, and also in honeybees in response to low developmental temperatures^86^. These observations raise new expectations to be tested in future studies in the field. For example, sampling adults of *D. buzzatii* during the winter-spring transition showing similar wing morphology abnormalities, it would provide indirect evidence that this species is capable of overwintering as larvae or pupae. Ovarioles number reached its maximum at 25 °C in *O. sulphurea*, but we found no differences between cacti at low temperatures (10 ± 6 °C). Likewise, wing length is significantly affected by diet at 25 °C, but not at 10 ± 6 °C. One possibility could be that nutritional assimilation processes at high temperatures could be limited by a shorter developmental time. In contrast, differences in nutritional composition and assimilation between cacti could be buffered by longer developmental times, like those attained at 10 ± 6 °C.

### Cold tolerance and fatty acid composition

Emerging adults reared in simulated winter conditions, similar to those found in the collection site of the flies, expressed a phenotype associated with enhanced cold tolerance. This consists of a greater abundance of unsaturated fatty acids and shorter chill coma recovery times, compared to flies grown at higher temperatures (25 °C or 25 ± 6 °C).

Principal component analysis revealed that flies reared at 10 ± 6 °C can be discriminated by higher values of C14:1 and C16:1 and lower values of SFAs, from those reared at 25 °C. Indeed, these samples have an average UFA/SFA ratio of 2.8, almost twice of those reared at 25 °C, regardless of temperature fluctuation. In addition, flies reared at 10 ± 6 °C have a UFA concentration of 73.5 ± 0.3%, which is higher than flies reared at 25 °C or 25 ± 6 °C in any diet. Such an increase of the UFA/SFA ratio in response to low developmental temperatures has been reported in other species of *Drosophila*^87^.

According to the homeoviscous adaptation hypothesis^19^, at low temperatures organisms can undergo a restructuring of the lipidic composition of cell membranes, such as higher unsaturation ratio of fatty acids, resulting in the conservation of membrane fluidity and functionality^18^. When exposed to 0 °C for 12 h, flies reared at 10 ± 6 °C recovered faster. CCRT is thought to be mechanistically linked to spreading depolarization due to loss of membrane fluidity at low temperatures^73,88^. As such, short CCRTs have been associated with high UFA/SFA ratio^89,90^. Here, we found that flies reared at low temperatures have significantly faster recoveries from chill-coma compared to flies reared at high temperatures (25 °C and 25 ± 6 °C). It is worth noting that flies were reared at low temperatures, but acclimated at 25 °C as adults for a week before CCRT assay and fed *ad-libitum*, and yet differences persist, suggesting a strong developmental acclimation effect. It is possible, however, that juveniles assimilated compounds other than unsaturated fatty acids found in cacti, such as sterols^91,92^ or some secondary metabolite like free amino acids, polyols or sugars^18,93^. Indeed, we showed that in *D. buzzatii* adult cold acclimation resulted in significant accumulation of several candidate cryoprotectants, including proline and glycerol^55^. Whether these processes are also taking place as a consequence of developmental acclimation remains to be explored.

### Diet-Temperature interplay

Responses across several traits point to a strong interplay between developmental diet and thermal regimes. Accordingly, despite working with only five isofemale lines, we observed crossings of the reaction norms, indicating genetic variation for plasticity in all traits. Furthermore, the models showed a significant effect of the line factor, indicating genetic variation across lines, even after almost a decade of laboratory inbreeding. Although the lines differ from one another, this does not rule out the possibility that the overall fitness observed may be lower than in outbred wild populations, as a consequence of inbreeding depression. Thus, the genetic variation captured within these five lines cannot be assumed as representative of the diversity present in the natural population from which they originated. For instance, while Kristensen et al., 2011 reported no effects of inbreeding on the capacity for adaptive developmental acclimation^94^, many *Drosophila* species exhibit marked inbreeding depression in basal cold resistance^94^. This limits the ecological implications of our study. Therefore, to fully interpret the relevance of the severe drop in viability at low temperatures, we must await for the results of similar experiments in newly-caught samples of wild populations.

Our study of fitness-related traits shows results that are consistent with expectations of Jensen’s inequality, which refers to a differential performance achieved by an organism between constant and fluctuating thermal regimes^95,96^ that is entirely dependent on the temperature at which fluctuations occur^40,41^. In general, daily thermal fluctuations seem to be positive to fitness-related traits at low temperatures (10 °C) and detrimental at high temperature (25 °C). However, this effect is heavily modulated by diet, favoring flies reared in the semi-natural diets (particularly its main host, *O. sulphurea*) over the laboratory diet. Cactophilic flies are adapted to exploit primarily rotten cacti, which are low in sugars as compared with other *Drosophila* that breed on sugar-rich fruits^97,98^. In this context, a high carbohydrate concentration diet, as the laboratory diet, has previously been found to be detrimental to fitness in another cactophilic fly, *Drosophila mojavensis*^99^. Considering it as a stressor, a high carbohydrate-diet could interact with other stressors during development, resulting in a synergistic negative effect over fitness^32,36^. As such, the interaction of high fluctuating temperatures and high-sugar diet could explain the low viability of flies reared on laboratory diet under the 25 ± 6 °C regime. However, flies reared on the laboratory diet have a better cold tolerance compared to those reared in semi-natural diets, but only at constant 25 °C, and no differences were found between flies reared in both cacti. This effect could be due to the high-sugar diet, which even though it reduces viability of cactophilic flies^99^, it may be improving cold tolerance, as it has been observed in other *Drosophila* species^28,100^.

Additionally, our findings show that *D. buzzatii* reared in its alternative host (*Trichocereus terscheckii*) had negative consequences on fitness, as predicted by the Preference-Performance Hypothesis^101,102^, but these results are also strongly modulated by temperature across traits. For instance, rearing in its primary host, *O. sulphurea*, resulted in a higher ovariole number but only at 25 °C. Similarly, rearing in this host resulted in shorter developmental times only at 25 °C and 25 ± 6 °C, but not at 10 ± 6 °C. Notably, we found viability differences between cactus, in favor of *O. sulphurea*, but only at low temperatures (i.e., 10 ± 6 °C).It is still unclear which nutritional value or components of cactus resulted beneficial to *D. buzzatii*. We can rule out calories as flies reared in *T. terscheckii* diet outperformed those grown in the Laboratory diet, and the latter has double the calories than the former (see Table S1). We could also exclude lipid composition because despite the laboratory diet containing 12.5 times fewer lipids than *O. sulphurea* diet, both exhibit a qualitatively similar fatty acid composition, with high concentrations of palmitic, linoleic, and stearic acids, which altogether account for 50% of the fatty acid fraction (see Table S3). However, metabolic assimilation does not directly correspond to nutritional composition of diets, and it could be modulated by different factors. For example, C14:1, which encompasses between 3 and 21% of the fatty acid fraction of fly samples, is absent in both diets (Table S3). Interestingly, larvae fed with the same diet have significantly different fatty acid profiles when reared at different thermal regimes, highlighting the temperature dependence of metabolic processes.

## Conclusion

Rising temperatures and its variance due to global warming will certainly have an effect on species distribution and phenology, as geographical range is linked with thermal tolerance^103^. We have shown that *D. buzzatii* can take advantage of low temperature fluctuations in combination with cactus diet, which in the context of climate change, could result in an expansion of its range towards higher latitudes and altitudes, as well as an earlier population recovery in spring. However, due to anthropic activity, Cactaceae are endangered in several places, such as the Altiplano and eastern Brazil^47^, which are key points of the distribution of *D. buzzatii* and other cactophilic flies, limiting the potential spread of these fly species. Once again, the complexity of climate change impacts on species fitness calls for models that incorporate the effects of several biotic and abiotic variables, as well as their interactions.

## Supplementary Information

Below is the link to the electronic supplementary material.

Supplementary Material 1

## Data Availability

The raw data can be accessed through the following link: 10.6084/m9.figshare.28829846.
