# Supplementary material for: Thermal variability and diet interaction as driver of developmental overwintering in Drosophila buzzatii
Source: Sci Rep. 2025 Nov 7;15:39141. doi: 10.1038/s41598-025-25689-8 (PMC12594894; doi:10.1038/s41598-025-25689-8)
Supplement: Supplementary file 1 — Supplementary Material 1 [file 41598_2025_25689_MOESM1_ESM.pdf]

11. Supplementary material

Fatty Acid Extraction and Quantification Methodology:

Approximately 3.5 to 10 mg of lipids were recovered. Fatty acid methyl esters (FAMEs) of total lipids were prepared by reaction with 4% HCl in CH_3OH at 70°C for 2 h. After cooling, a drop of water was added and the FAMEs were extracted with 0.5 mL of dichloromethane three times. The organic phase containing FAMEs were analyzed by gas chromatography (CG-FID) on a Focus GC (Thermo Finnigan Corporation), equipped with an Innowax capillary column (Agilent, 100% polyethylene glycol, 30 m length, 0.25 mm i.d., 0.5 µm film thickness). Nitrogen was used as carrier gas (90 kPa constant pressure). Manual injection was operated in splitless mode at 240°C and detector temperature was set at 300°C. The temperature program was 100°C (1 min), 15 °C/min to 200°C (1 min), and 2°C/min to 240°C (18 min). Individual FAMEs were identified by comparing their retention times with those of authentic laboratory standards (Sigma-Aldrich Co.). Quantification was performed by comparing the peak area percentages of each FAME on the chromatogram with that of the internal standard of known weight (methyl nonadecanoate, Sigma-Aldrich Co.). Individual FAMEs were expressed as a percentage of the total fatty acids. Along with the individuals, fatty acid compositions of the experimental diets were also analyzed.

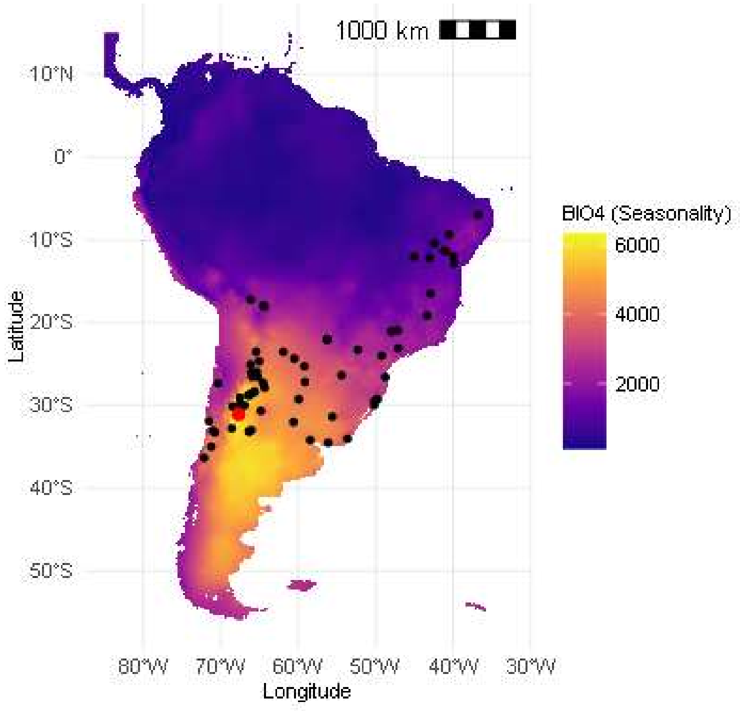

Figure S1: Distribution of *D. buzzatii* over the South American continent. Black dots represent locations where *D. buzzatii* is found [53]. In red, the collecting site (San Agustín del Valle Fértil, San Juan, Argentina). Color backgrounds represent values of temperature seasonality (Bioclimatic variable 4 in WorldClim), defined as 100 times the standard deviation of annual temperature [104].

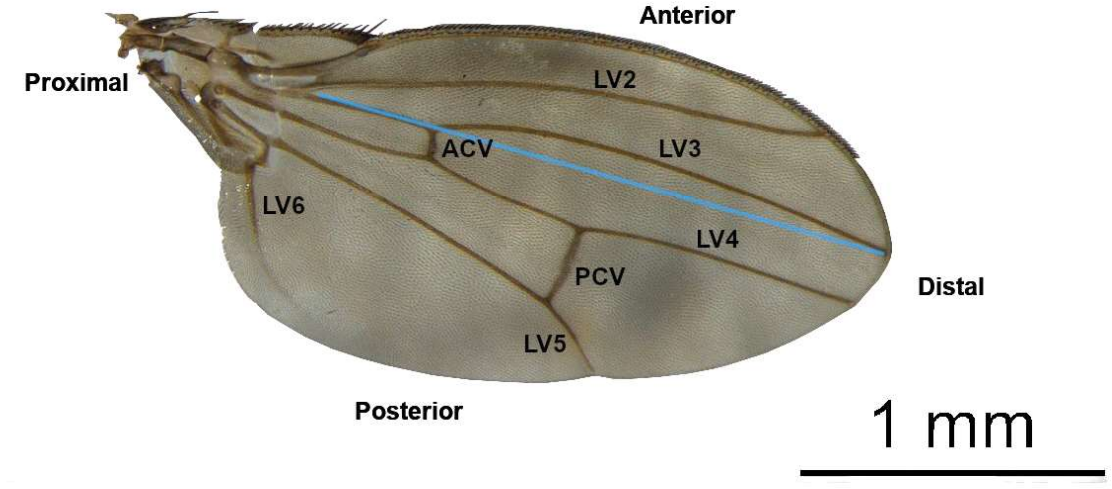

Figure S2: Dorsal view of a female wing of *Drosophila buzzatii*. Wing length was measured as the distance in millimeters between the beginning and the end of vein 3 (blue line).

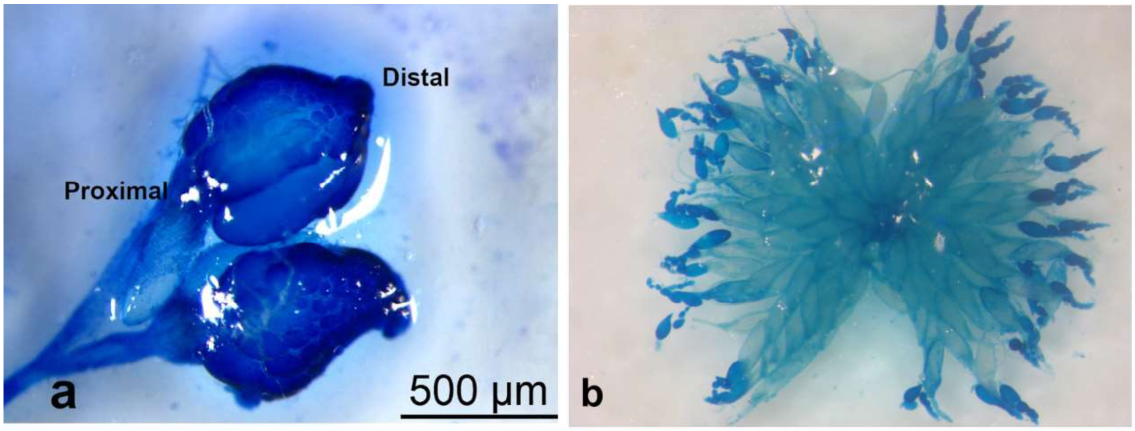

Figure S3: Ovaries of *Drosophila buzzatii* dyed with methylene blue. **a**: Whole intact ovaries. **b**: Dissected ovaries, with individual ovarioles separated and extended. Germaria and immature oocytes at the distal extreme of each ovariole are darker.

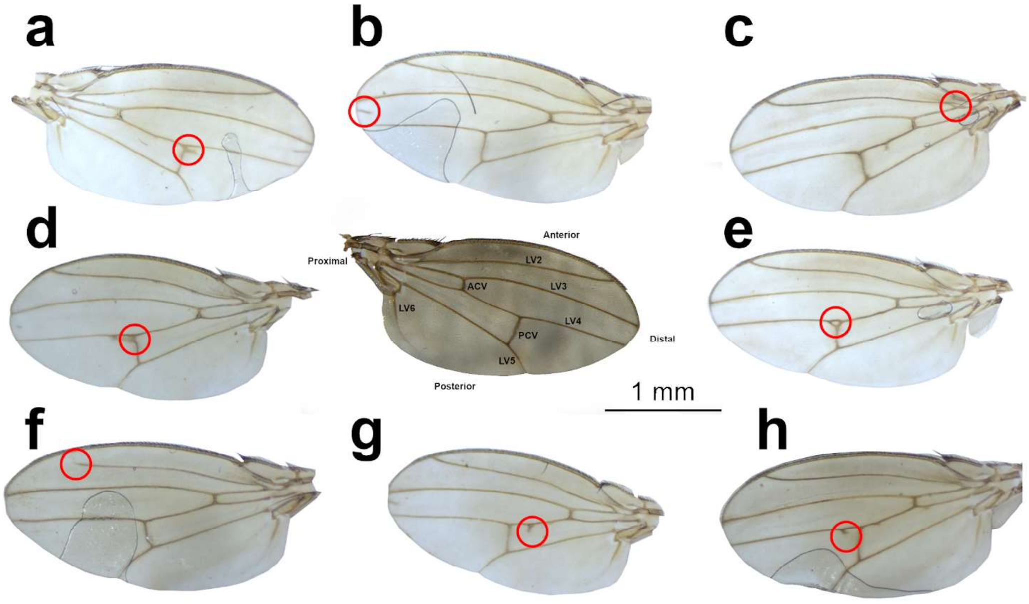

Figure S4: Examples of the malformations observed in *D. buzzatii* wings reared on cacti (*O. sulphurea* or *T. terscheckii*) at 10±6°C. **a:** Thickening at the union of LV4 and the ACV. **b:** Incipient ectopic vein between LV3 and LV4 distally. **c:** Malformation at the beginning of LV2. **d:** Broken PCV vein. **e:** Malformation at the union of LV4 and the ACV. **f:** Broken LV2 vein. g: Incipient ectopic vein anteriorly on the proximal side of the PCV vein. **h:** Incipient ectopic vein anteriorly on the distal side of the PCV vein. Center: a normal *D. buzzatii* wing.

Table S1: Nutritional composition of experimental diets.

Diet (c/100g)	Water (g)	Carbohydrates (g)	Protein (g)	P:C	Lipids (g)	Kcal
O. sulphurea [a]	89.90	3.22	0.74	1:4	0.50	35.05
T. terscheckii [b]	95.40	1.20	0.70	1:2	0.20	15.25
Laboratory [c]	~90	7.33	0.78	1:9	0.04	32.86

a: Carreira et al., 2014 [96]. b: Padró & Soto, 2013 [97]. c: Flaibani et al., 2023 [103].

Table S2: Average fatty acid composition by experimental group.

Diet	Laboratory		*O. sulphurea*		
Temperature (°C)	25	25±6	25	25±6	10±6
C10:0 (Capric acid	0.52	0.50	0.50	0.53	0.31
C12:0 (Lauric acid)	2.04	2.33	2.22	2.13	1.69
C12:1 (Lauroleic acid)	0.35	0.41	0.64	0.61	0.62

C14:0 (Myristic acid)	19.75	21.03	18.66	17.67	11.35
C14:1 (Myristoleic acid)	5.46	4.68	3.57	3.30	12.59
C16:0 (Palmitic acid)	18.30	17.99	18.82	19.32	12.59
C16:1 (Palmitoleic acid)	27.29	28.50	26.01	26.25	32.21
C18:0 (Stearic acid)	0.87	0.93	1.05	1.21	0.52
C18:1 (Oleic acid)	21.13	19.99	21.15	22.05	22.67
C18:2 (Linoleic acid (ω-6))	2.73	2.53	5.52	5.17	3.60
C18:3 (α-Linolenic acid (ω-3))	1.55	1.06	1.76	1.60	1.83
SFA	41.50	42.84	41.34	41.02	26.48
UFA	58.50	57.16	58.66	58.98	73.52
PUFA	4.28	3.58	7.29	6.77	5.44
MUFA	54.22	53.57	51.37	52.21	68.09
UFA/SFA ratio	1.41	1.34	1.42	1.44	2.78

Values represent the percentage of every fatty acid over total fatty acid fraction.

Table S3: Average fatty acid composition of experimental diets.

Diet	Laboratory	*O. sulphurea*
C10:0 (Capric acid)	1.37	0.52
C12:0 (Lauric acid)	0.48	1.39
C14:0 (Myristic acid)	0.00	1.50
C16:0 (Palmitic acid)	25.34	30.47
C16:1 (Palmitoleic acid)	9.82	5.93
C17:0 (Margaric acid)	0.00	1.24
C18:0 (Stearic acid)	14.69	15.98
C18:1 (Oleic acid)	11.16	11.72

C18:2 (Linoleic acid (ω-6))	29.22	21.64
C18:3 (α-Linolenic acid (ω-3))	7.60	8.10
C20:0 (arachidic acid)	0.32	1.51

Values represent the percentage of every fatty acid over total fatty acid fraction.

Table S4: Number of replicates (n) used in the experimental assays.

Temperature	Diet	Isoline	Viability	Developmental time	Ovariole number	Wing length	Chill coma recovery time
10	Laboratory	DB101	16	0	0	0	0
10	Laboratory	DB23	16	0	0	0	0
10	Laboratory	DB36	16	0	0	0	0
10	Laboratory	DB44	16	0	0	0	0
10	Laboratory	DB60	16	0	0	0	0
10±6	Laboratory	DB101	16	0	0	0	0
10±6	Laboratory	DB23	16	0	0	0	0
10±6	Laboratory	DB36	16	0	0	0	0
10±6	Laboratory	DB44	16	0	0	0	0
10±6	Laboratory	DB60	16	0	0	0	0
25	Laboratory	DB101	10	40	6	23	0
25	Laboratory	DB23	14	69	3	44	0
25	Laboratory	DB36	15	32	11	44	0
25	Laboratory	DB44	9	21	13	59	0
25	Laboratory	DB60	11	33	4	21	0
25±6	Laboratory	DB101	8	27	9	47	0
25±6	Laboratory	DB23	12	41	12	68	0
25±6	Laboratory	DB36	12	54	18	89	0
25±6	Laboratory	DB44	8	16	5	22	0

25±6	Laboratory	DB60	8	21	8	32	0
25	Laboratory	DB101	0	0	0	0	26
25	Laboratory	DB23	0	0	0	0	36
25	Laboratory	DB36	0	0	0	0	40
25	Laboratory	DB60	0	0	0	0	24
25±6	Laboratory	DB101	0	0	0	0	32
25±6	Laboratory	DB23	0	0	0	0	24
25±6	Laboratory	DB36	0	0	0	0	37
25±6	Laboratory	DB44	0	0	0	0	18
25±6	Laboratory	DB60	0	0	0	0	28
10	*O. sulphurea*	DB101	16	0	0	0	0
10	*O. sulphurea*	DB23	16	0	0	0	0
10	*O. sulphurea*	DB36	16	0	0	0	0
10	*O. sulphurea*	DB44	16	0	0	0	0
10	*O. sulphurea*	DB60	16	0	0	0	0
10±6	*O. sulphurea*	DB101	16	32	9	10	12
10±6	*O. sulphurea*	DB23	16	45	1	0	6
10±6	*O. sulphurea*	DB36	16	61	9	10	12
10±6	*O. sulphurea*	DB44	16	60	10	18	22
10±6	*O. sulphurea*	DB60	16	57	10	23	25
25	*O. sulphurea*	DB101	10	63	10	61	41
25	*O. sulphurea*	DB23	9	51	10	48	52
25	*O. sulphurea*	DB36	8	48	11	25	40
25	*O. sulphurea*	DB44	6	47	10	28	38
25	*O. sulphurea*	DB60	4	41	11	38	32
25±6	*O. sulphurea*	DB101	14	43	7	56	49
25±6	*O. sulphurea*	DB23	15	55	10	86	74

25±6	*O. sulphurea*	DB36	6	50	9	52	49
25±6	*O. sulphurea*	DB44	8	33	10	29	33
25±6	*O. sulphurea*	DB60	14	49	10	45	48
10	*T. terscheckii*	DB101	14	0	0	0	0
10	*T. terscheckii*	DB23	14	0	0	0	0
10	*T. terscheckii*	DB36	14	0	0	0	0
10	*T. terscheckii*	DB44	14	0	0	0	0
10	*T. terscheckii*	DB60	14	0	0	0	0
10±6	*T. terscheckii*	DB101	8	21	1	1	1
10±6	*T. terscheckii*	DB36	8	21	3	1	2
10±6	*T. terscheckii*	DB44	8	61	10	12	13
10±6	*T. terscheckii*	DB60	8	12	6	5	6
10±6	*T. terscheckii*	DB23	8	6	0	0	0
25	*T. terscheckii*	DB101	8	80	11	30	31
25	*T. terscheckii*	DB23	8	24	10	29	31
25	*T. terscheckii*	DB36	8	98	10	35	36
25	*T. terscheckii*	DB44	8	93	10	17	32
25	*T. terscheckii*	DB60	8	58	10	33	34
25±6	*T. terscheckii*	DB101	8	38	13	35	27
25±6	*T. terscheckii*	DB23	8	40	10	36	37
25±6	*T. terscheckii*	DB36	8	36	11	34	36
25±6	*T. terscheckii*	DB44	8	40	10	35	36
25±6	*T. terscheckii*	DB60	8	43	10	34	35

Table S5: Mean, median and standard deviation of each experimental group.

	Diet	Thermal regime (°C)	Mean	Median	SD
Ovariole number	Laboratory	25	30.92	31.00	7.91

	Laboratory	25±6	32.18	32.00	5.81
	O. sulphurea	10±6	33.41	33.00	10.89
	O. sulphurea	25	40.00	41.00	5.63
	O. sulphurea	25±6	33.63	35.50	7.63
	T. terscheckii	10±6	28.70	27.50	9.66
	T. terscheckii	25	33.24	32.00	6.86
	T. terscheckii	25±6	30.94	31.50	7.90
Wing length (mm)	Laboratory	25	1.89	1.88	0.09
	Laboratory	25±6	1.84	1.85	0.13
	O. sulphurea	10±6	2.12	2.13	0.07
	O. sulphurea	25	2.01	2.04	0.09
	O. sulphurea	25±6	2.02	2.04	0.08
	T. terscheckii	10±6	2.08	2.10	0.07
	T. terscheckii	25	2.03	2.03	0.03
	T. terscheckii	25±6	2.03	2.04	0.04
Viability (%)	Laboratory	10	0.00	0.00	0.00
	Laboratory	10±6	0.00	0.00	0.00
	Laboratory	25	28.26	25.00	17.00
	Laboratory	25±6	15.68	15.00	11.48
	O. sulphurea	10	0.00	0.00	0.00
	O. sulphurea	10±6	10.38	5.00	13.33
	O. sulphurea	25	55.61	60.00	25.63
	O. sulphurea	25±6	59.12	62.50	18.60

	T. terscheckii	10	0.00	0.00	0.00
	T. terscheckii	10±6	2.00	0.00	3.76
	T. terscheckii	25	41.44	47.50	28.08
	T. terscheckii	25±6	62.00	65.00	21.96
Developmental time (days)	Laboratory	25	13.69	13.00	2.33
	Laboratory	25±6	14.08	14.00	1.59
	O. sulphurea	10±6	91.06	90.00	6.04
	O. sulphurea	25	11.33	11.00	1.71
	O. sulphurea	25±6	12.51	12.00	1.44
	T. terscheckii	10±6	92.45	92.00	7.25
	T. terscheckii	25	12.24	12.00	1.70
	T. terscheckii	25±6	13.12	13.00	1.28
Chill-coma recovery time (sec)	Laboratory	25	1594.57	1510.00	650.26
	Laboratory	25±6	1567.55	1365.50	754.48
	O. sulphurea	10±6	1078.78	840.00	743.07
	O. sulphurea	25	1998.38	1709.00	1036.50
	O. sulphurea	25±6	1803.74	1500.00	899.84
	T. terscheckii	10±6	859.09	780.00	538.69
	T. terscheckii	25	1879.02	1680.00	823.53
	T. terscheckii	25±6	1689.12	1320.00	924.06

Table S6: GLMM analysis of viability at 25°C and 25±6°C.

Factor	$\chi 2$	Degrees of freedom	p-Value

Diet	108.28	2	<0.0001
Daily fluctuation	2.83	1	0.0927
Diet-Daily Fluctuation Interaction	57.52	2	<0.0001

Analysis of variance of the model used for the high temperature (25°C and 25±6°C) viability subset shows a significant Diet-by-Daily thermal fluctuation interaction. For this analysis, data was fitted to a Beta-Binomial distribution.
